# On the Vertigo Due to Static Magnetic Fields

**DOI:** 10.1371/journal.pone.0078748

**Published:** 2013-10-30

**Authors:** Omar S. Mian, Yan Li, Andre Antunes, Paul M. Glover, Brian L. Day

**Affiliations:** 1 Sobell Department of Motor Neuroscience and Movement Disorders, Institute of Neurology, University College London, London, United Kingdom; 2 Sir Peter Mansfield Magnetic Resonance Centre, University of Nottingham, Nottingham, United Kingdom; Hospital Nacional de Parapléjicos, Spain

## Abstract

Vertigo is sometimes experienced in and around MRI scanners. Mechanisms involving stimulation of the vestibular system by movement in magnetic fields or magnetic field spatial gradients have been proposed. However, it was recently shown that vestibular-dependent ocular nystagmus is evoked when stationary in homogenous static magnetic fields. The proposed mechanism involves Lorentz forces acting on endolymph to deflect semicircular canal (SCC) cupulae. To investigate whether vertigo arises from a similar mechanism we recorded qualitative and quantitative aspects of vertigo and 2D eye movements from supine healthy adults (n = 25) deprived of vision while pushed into the 7T static field of an MRI scanner. Exposures were variable and included up to 135s stationary at 7T. Nystagmus was mainly horizontal, persisted during long-exposures with partial decline, and reversed upon withdrawal. The dominant vertiginous perception with the head facing up was rotation in the horizontal plane (85% incidence) with a consistent direction across participants. With the head turned 90 degrees in yaw the perception did not transform into equivalent vertical plane rotation, indicating a context-dependency of the perception. During long exposures, illusory rotation lasted on average 50 s, including 42 s whilst stationary at 7T. Upon withdrawal, perception re-emerged and reversed, lasting on average 30 s. Onset fields for nystagmus and perception were significantly correlated (p<.05). Although perception did not persist as long as nystagmus, this is a known feature of continuous SSC stimulation. These observations, and others in the paper, are compatible with magnetic-field evoked-vertigo and nystagmus sharing a common mechanism. With this interpretation, response decay and reversal upon withdrawal from the field, are due to adaptation to continuous vestibular input. Although the study does not entirely exclude the possibility of mechanisms involving transient vestibular stimulation during movement in and out of the bore, we argue these are less likely.

## Introduction

Vertigo, the non-veridical perception of motion of self or surroundings, is sometimes experienced by patients and operators in MRI environments [1]–[3]. A number of mechanisms by which magnetic fields may induce vertigo via stimulation of the vestibular system were considered by Glover et al [4]. The mechanisms depended on movement through magnetic fields or magnetic fields with high spatial gradients, and human-based studies have lent some support to such factors being important [1], [2], [4]–[6]. In contrast, it was recently demonstrated that static homogenous magnetic fields are capable of stimulating the stationary vestibular system in humans to evoke ocular nystagmus [7]. It was proposed that magnetic fields interact with spontaneous ionic current flow in labyrinthine endolymph to induce Lorentz forces strong enough to deflect semicircular canal cupulae. In the same study, the authors also mentioned that *“most subjects reported a sense of rotation, usually after they were completely in the bore and the table stopped moving. This sensation often died away after a minute or so”*. This prolonged perceptual response suggests that, like ocular nystagmus, magnetic field-evoked vertigo does not necessarily depend on movement or gradient magnetic fields. However, no further details of the evoked perceptual responses were reported.

The current study was designed to provide a more comprehensive understanding of the properties of magnetic field-evoked vertigo. To achieve this we recorded qualitative and quantitative aspects of the vertigo and ocular nystagmus experienced by healthy participants when pushed into the static magnetic field of a 7T MRI scanner. Our aims were to record unbiased descriptions of vertigo, and then to determine onset field strengths, magnitudes, plane and direction of the illusory motion perceptions with respect to the magnetic field and gravity. With these data we could consider the compatibility between magnetic-evoked vertigo and the hypotheses that have been put forward for magnetic-evoked vestibular stimulation, as well as its congruence with magnetic-evoked nystagmus.

## Methods

The study was performed at the University of Nottingham. The participant pool upon which this paper is based (n = 25) had an age range of 18–60 years (mean±SD = 29±10) and was 48% male. All participants were screened by questionnaire to exclude those with general contraindications for the MRI environment, as well as those with neurological or otological conditions and those who suffer from claustrophobia.

### Ethics statement

The study received approval from Medical School Research Ethics Committee of the University of Nottingham. All participants provided signed, informed consent.

### General procedures

**Figure 1:**
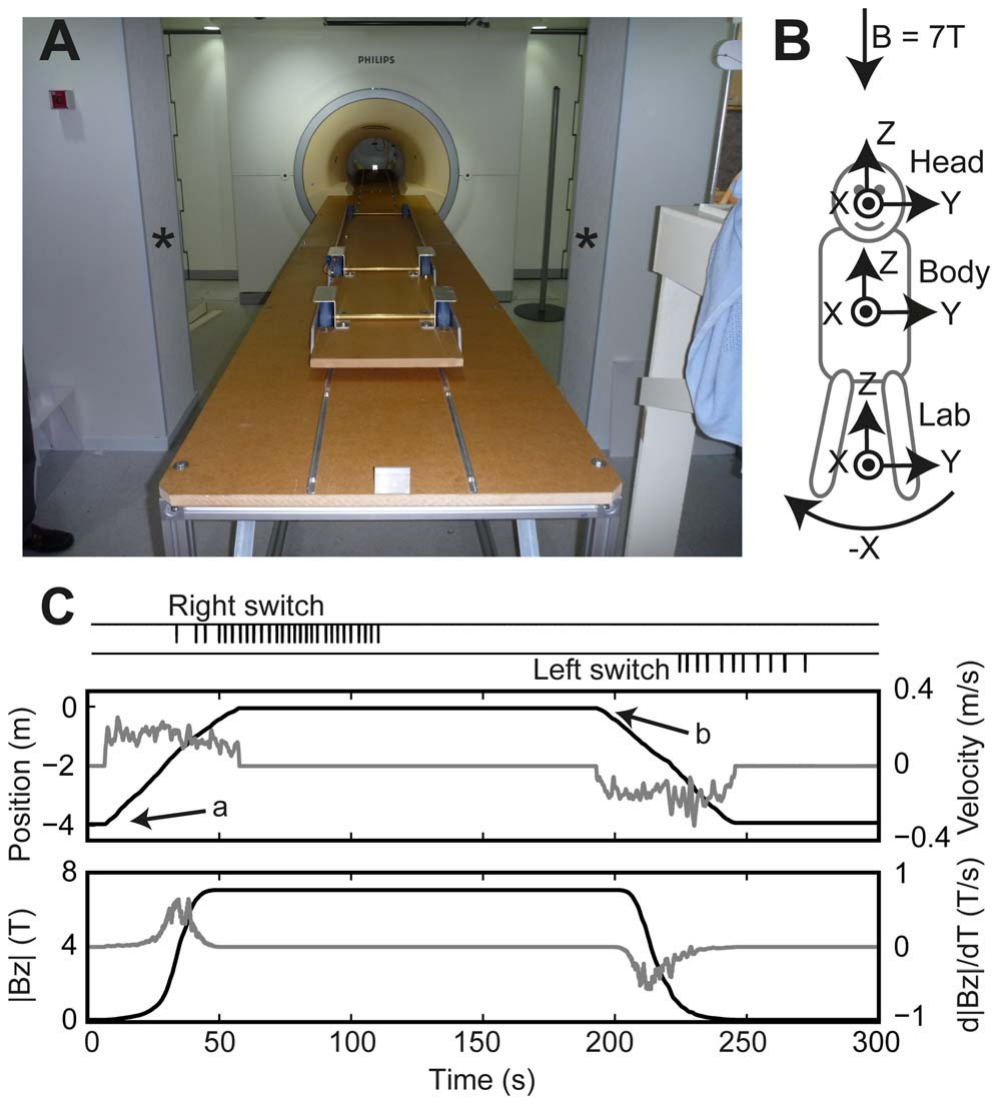
Experimental setup. **A)** Photograph of custom-made track and bed in scanner room. The head started between the two asterisks, approximately 2.4 m from the entrance to the bore and 4 m to the centre of the bore. **B)** Head, body, and lab coordinate systems viewed from above participant lying on table. X-axes point out of page. For the head, the XY plane is in Reid's plane (the plane formed by the external auditory meati and the lower orbital margins). Rotation polarities are described according to the right-hand grip-rule (e.g. curved arrow depicts negative rotation direction about the X-axes). We also refer to head or trunk rotations about their X,Y, and Z axes as roll, pitch, and yaw respectively. When entering magnet head first, the direction of the static magnetic field (B) was in the head-to-toe (-Z) direction. The origin of the lab coordinate system is at the centre of the bore for the purpose of linear position descriptions. **C)** Example data from a long-duration exposure of experiment 2. Middle panel shows head position (black, left axes) and velocity (grey, right axes) along lab Z axis. Bottom panel shows magnetic field magnitude (black, left axes) and its temporal rate of change (grey, right axes) experienced by the head along the lab Z axis. Top panel shows raw voltage output from switches for logging perceived rotation (downward pulses depict depression of switches). We refer to the portion of the trial between the start of movement into the scanner (time a) and start of movement out of the scanner (time b) as the in-phase. Everything after time b is the out-phase.

Experiments were conducted in a 7 T Philips Achieva MRI scanner. No imaging was performed, so only the static magnetic field was present. A custom-made track and bed was installed in place of the standard Philips bed (Figure 1A). This was deemed important for two reasons: 1) Smooth castors and tracks were used to minimise jerkiness of bed motion into and out of the bore. This, together with exclusion of vision, served to minimise veridical motion information during the experiments; 2) the frame was constructed to the maximum length possible in the scanner room. This meant that the baseline field exposure of the head at the start of each trial could be much lower (approx. 0.1T) than usual (approx. 1T).

Three experiments were performed, separated by several weeks. The general aims were to obtain qualitative and/or quantitative data on induced vertiginous perceptions, and sometimes eye movements, when participants were exposed to the magnetic field of the bore. Most of the data presented in this paper were obtained with the participants lying in a supine position with the orientation of Reid's plane (the plane formed by the external auditory meatus and the lower orbital margins) approximately vertical and with head first entry into the scanner (experiment 3 is the exception). All trials were performed without vision. For some participants in experiment 2 this was achieved by eye closure. Otherwise, trials were performed with eyes open in total darkness to enable recording of eye movements. Darkness was achieved by turning off room lights and blocking visible light sources. To aid this, a felt cloth draped over a frame formed a tent around the participants head and the eye tracking equipment. All participants confirmed this achieved complete darkness.

Prior to the start of each trial, the head was guided to the desired orientation. Padding and wedges were used for comfort and stability. Photos were then taken using digital cameras mounted on the wall and ceiling of the scanner room in order to measure orientation and start position within the room. Vision was then removed (eyes closed or room made dark) and participants remained stationary on the bed until the end of the trial. During the trial the experimenter manually pushed the bed into the bore. In addition to any perceptual reports provided during trials, at the end of each trial participants were asked to sit up and describe their perceptions, using a doll to assist their descriptions of perceived motion.

### Coordinate systems and magnetic field

To aid description of the experiments and results we refer to lab, body (trunk), and head right-hand coordinate systems (Figure 1B). With the body supine with Reid's plane vertical and for head-first entry into the bore (approximately the standard position for head scans), body-relative-to-lab, head-relative-to-lab, and head-relative-to-body XYZ angles are all zero. For the head and body, we use the general terms roll, pitch, and yaw to refer to rotations about X, Y, and Z axes respectively. The polarities of rotations are reported according to the right-hand grip-rule. Thus, with the subject supine and a neutral head position, a perceived horizontal plane rotation may be described as a roll. If the perceived direction involves legs rotating to the right the polarity is reported –X direction of rotation in all reference frames (Figure 1B). Horizontal eye movements refer to ocular rotations about the ocular axis parallel with head Z (leftward = +Z; rightward = −Z) and vertical eye movements refer to ocular rotations about the ocular axis parallel with head Y (downward = +Y; upward = −Y).

When participants are pushed into the scanner magnet bore, the strength of the static magnetic field experienced by the head gradually increases (Fig 1C). Thus, it experiences both a spatially-varying (usually referred to as a magnetic field gradient) and a temporally-varying (dB/dt) magnetic field (due to velocity through the gradient) only during entry and exit. The point of maximum gradients, and hence dB/dt, is near to the entrance to the bore. At the iso-centre of the scanner magnet the magnetic field is 7T and points in the linear –Z direction (i.e. no X and Y components). This is head-to-toe for head first entry. It is homogenous over a +/−30 cm range in the Z direction. Away from the iso-centre of the scanner, and off axis, the vestibular system may experience X and Y components. Given the vestibular labyrinths are no more than 10 cm lateral to the Z axis in the current experiment, we estimate these components should not exceed 0.5T and would only occur at the points of highest magnetic field gradient.

### Recording devices

An optical encoder (HEDS-5701-F00, Hewlett Packard) coupled to one of the wheels of the bed was used to record the movement of the bed. By combining the starting position of the head in room coordinates with the bed displacement data, we could localise the position of the head in room coordinates throughout the trial. The magnetic field experienced by the head in the Z direction (Bz) was then determined using the magnetic field profile of the MRI room provided by Philips. In experiment 2 and 3, participants used air-operated switches to log their perception of rotation (Fig 1C). Analog bed encoder and switch signals were captured onto a PC at 5 kHz via a data acquisition card (USB-6009, National Instruments).

To record eye movements, an infra-red (IR) camera was mounted on a frame above the participants' left eye. This camera was a modified webcam (V-U0012 Logitech, Romanel-sur-Morges, Switzerland) with components removed and housed in a plastic box. The lens barrel of the camera was temporarily removed and the IR-cut filter removed. This extended the camera's sensitivity into the near IR. Two IR (920 nm) LEDs (HIRL5040, Rodan, Taichung, Taiwan) were used for illumination of the eye. Recordings were 5-10 fps in experiment 1 and 25-30 fps in experiment 2. The low frame rate in experiment 1 was inadvertent, but was sufficient for visual inspection of the eye movements. Participants wore a pair of glasses with large high contrast frames which provided an initial spatial reference for pupil tracking.

A microphone mounted on the bed was used to record any qualitative descriptions of perceptions given during and after trials. An audible beep at the start of acquisition by the data acquisition card was used to align these data with audio and video data. Detection of precise timing of audio events was achieved with the aid of software to display the audio waveform.

### Experiment 1

**Figure 2:**
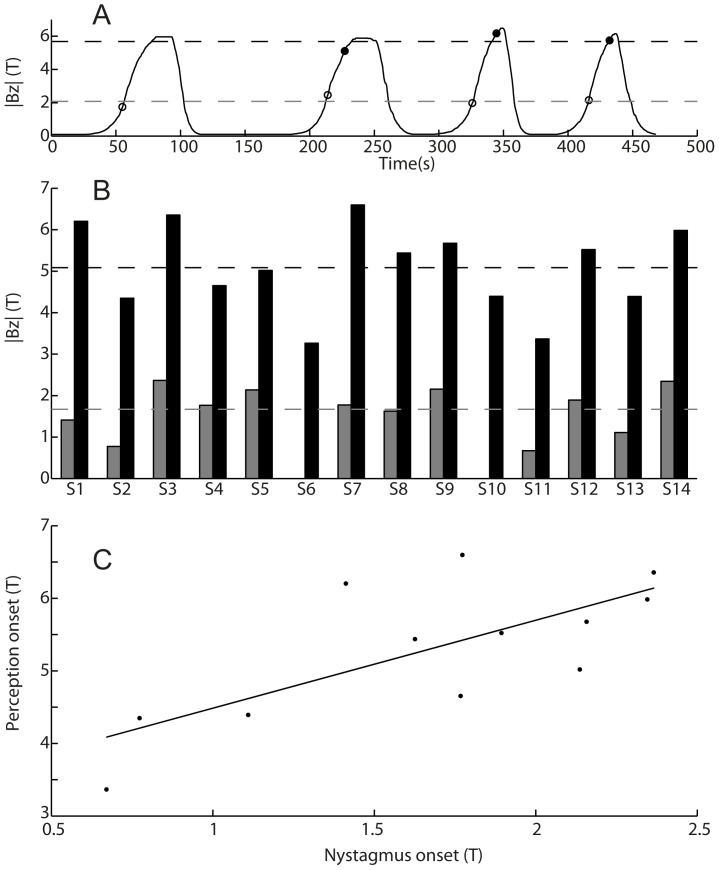
Thresholds of nystagmus and motion perception. **A)** Data from single participant showing magnitude of the Z magnetic field (Bz) experienced by the head due to bed being pushed into and removed from the bore 4 times with overlayed open circles indicating points at which nystagmus onset was detected and solid circles indicating points at which onset of non-veridical motion perception was reported for each entry. There is no solid circle for the first entry because this is used as a perceptual scout entry (refer to main text). Horizontal lines represent mean across entries. **B)** Onset field for nystagmus (grey bars) and perception (black bars) for each participant and group means (horizontal lines). Nystagmus onset not obtained for S6 or S10. **C)** Perception onset plotted against nystagmus onset with linear regression line. Pearson correlation coefficient = 0.72 (p<.01).

Fourteen participants took part in experiment 1 which had two purposes. First, to obtain unbiased descriptions of the perceptions experienced in participants who had not previously been pushed into a 7T magnetic field in darkness. Second, to establish the field strength at which the onset of eye movements and vertigo occurred during entry. The participants were told that people occasionally experience unusual perceptions when inside an MRI scanner, but the nature and strength of perception can vary from person to person, and so the purpose of the experiment was to understand the incidence and characteristics of any such perceptions. After the initial setup, the experiment lasted about 10 minutes. Participants were exposed to 3–4 entries into the scanner (e.g. Fig 2A). Each entry involved being moved slowly into the scanner, left inside for a few seconds, and then slowly withdrawn. Participants were pushed only a little beyond the point at which they indicated onset of vertiginous perception and then removed shortly after. There was a break of approximately 30–60 s separating each entry. The entire experiment (including breaks) was performed with eyes open in darkness, with the camera recording the eyes. The task of the participants was to verbalise their perceptual experiences as they were occurring. The first entry served as a scout, in which participants gave naïve and unbiased descriptions of their perceptions. In subsequent entries, they were asked to give a clear verbal indication (i.e. say “now”) as soon as these perceptions (and/or any different perceptions) started. Based on the initial descriptions given by each participant during the experiment, we also asked them to decide whether or not they could further refine their descriptions in subsequent entries (for example, if a participant initially described a perception of spinning, we asked them to determine if they could discern the direction they were spinning in subsequent entries). Also, if participants gave ambiguous descriptions such as dizziness, we sought clarification as to whether or not this meant they had a perception of motion.

The audio and video were reviewed offline. Onset of horizontal nystagmus was determined by visual inspection of the recorded videos. The first nystagmus quick phase with horizontal component was identified and then the onset of nystagmus was defined as the beginning of the preceding slow phase as judged by stepping frame by frame through the video. The mean of the independent judgements of two of the authors was used. The waveform of the recorded audio was used to establish exactly when the onset of vertiginous perception was reported.

### Experiment 2

Nineteen volunteers took part in experiment 2 (9 of whom had participated in experiment 1). The most common perceptual experience reported in experiment 1 was one of rotation in the horizontal plane. The main purpose of experiment 2 was to attempt to quantify the time course, magnitude, and direction of this perception as participants were slowly pushed into the field, remained stationary inside the central homogenous field for an extended time period, and then removed from the field. Removal of vision was achieved by eye closure in some participants and by darkened environment in others.

The bed was slowly pushed into the magnet with the aim of covering the approximately 4 m distance from the start position to the centre of the magnet in approximately 50–60 s. A wall mounted stopwatch was used to aid the experimenter. Once stationary, the participant was kept at the centre of the magnet for 135 s and then returned to the start position at approximately the same rate as the rate of entry (see Figure 1C). Pilot tests had revealed that a dwell time of 135 s should allow capture of the full course of the perceptual response. Participants were asked to focus their attention on perceptions of horizontal plane rotation. To quantify the speed of perceived horizontal plane rotation, we used an approach similar to that used in perceptual studies of physical rotation stimulation [e.g. 8]. Participants held an air operated switch in each hand. Each time they perceived themselves rotating through a prescribed angle, they would press a switch (e.g. Fig 1C). This angle was typically 45 deg, but we allowed the participants to use a different angle if they felt it would be easier. Using the time interval between switch presses, we calculated a perceived velocity of rotation. The direction of rotation was indicated by the switch that was pressed. One to three practice trials were performed prior to recorded trials.

Automated pupil tracking was performed offline using in-house written software. The algorithm consisted of measuring the centre of the pupil within the coordinate frame provided by the glasses. This was achieved by calculating the average centre of ellipses constructed from points in the high contrast border between the pupil and iris. Pupil displacements were transformed from glasses frame to anatomical coordinates (horizontal axis = inter-aural axis; vertical axis perpendicular to Reid's plane) and then converted to eye rotations (see earlier coordinate systems section) using the assumption of a 12 mm eyeball radius, which exhibits little variation between adults [9]. The slow-phase velocity was then determined from the slow drift of the eye angle between nystagmus quick phases. In many cases, eye movement recordings were obtained on separate trials to those used for perceptual reporting.

### Experiment 3

Six participants who had taken part in experiment 2 were studied. Of the participants available on the day of experiment 3, they were the six we considered most able to give lucid descriptions of perception. The purpose was to examine the influence of static head yaw orientation on the perceptions evoked by exposure to the magnetic field. Trial duration and bed motion was the same as in experiment 2 and trials were performed with eyes open in darkness. With participants supine, three head yaw orientations (target head angles: neutral, 90 deg left yaw, 90 deg right yaw) were tested in a random order. At the start of each trial we used a hand-held goniometer to record attained head yaw orientations.

During three initial trials (one at each head orientation), participants did not use switches to report horizontal plane rotation in order to avoid priming of attention to this plane. Instead, they freely described any perceptions they experienced during the trial. Following these trials, the conditions were repeated with the switches to log perception of horizontal plane rotation, as for experiment 2.

### Statistical analysis

Unless otherwise stated, group data are reported as mean±SD. Statistical comparisons, between the in-phase and out-phase, of bed movement characteristics (experiments 2 and 3) and duration and magnitude of perception (experiment 2) were made using repeated measures t-tests (2-tailed). Comparisons between onset of vertigo in experiment 1 and 2 were made using repeated measures t-test (2-tailed). Comparisons between head orientation conditions in experiment 3 were made using Friedman's non-parametric ANOVA. Analysis of the relationship between onset of vertigo and nystagmus was made using Pearson's product moment correlation coefficient (1-tailed; experiment 1).

## Results

The initial sections report data obtained at the neutral head position. The final section reports on the effect of head yaw orientation.

### Bed movement, field exposure, and head positions

Typical bed magnetic field profiles experienced during the short exposures of experiment 1 and the long exposures of experiments 2 and 3 can be seen in Figure 2A and 1C respectively. Group summaries of recorded head pitch orientations, and bed movement speed and magnetic field exposure measures are shown in table 1. As intended, during long exposure trials the entry and exit bed movement were similar. However, mean speed and peak dB/dt (experiment 2 only) tended to be slightly but significantly higher during exit (P<.05). This is attributed to use of manual, rather than motorized bed motion.

**Table 1:** Bed movement, magnetic field exposure characteristics, and head pitch orientations for entries during neutral head position trials.

	Experiment 1	Experiment 2	Experiment 3
	(short-duration exposure)	(long-duration exposure)	(long-duration exposure)
N	14	19	6
Head pitch orientation (deg)	−10±5	−6±7	−7±3
Mean speed (m/s), entry	0.05±0.01	0.069±0.014	0.068±0.009
Mean speed (m/s), exit	N/A	0.079±0.016 **	0.077±0.012 *
Peak |dB_Z_|/dt (T/s), entry	0.40±0.05	0.63±0.14	0.61±0.11
Peak |dB_Z_|/dt (T/s), exit	N/A	0.69±0.16 *	0.61±0.12
Duration dB_Z_/dt exposure (s), entry	27±5	21±4	21±3
Duration dB_Z_/dt exposure (s), exit	N/A	20±4	21±5
Duration stationary at centre of magnet (s)	N/A	136±6	137±5

Duration of dBz/dt exposure was arbitrarily defined as the duration the head was experiencing dBz/dt magnitude greater than 0.1. Comparisons between entry and exit: * P<.05, ** P<.01

### Qualitative characteristics of eye movements and perception

**Figure 3:**
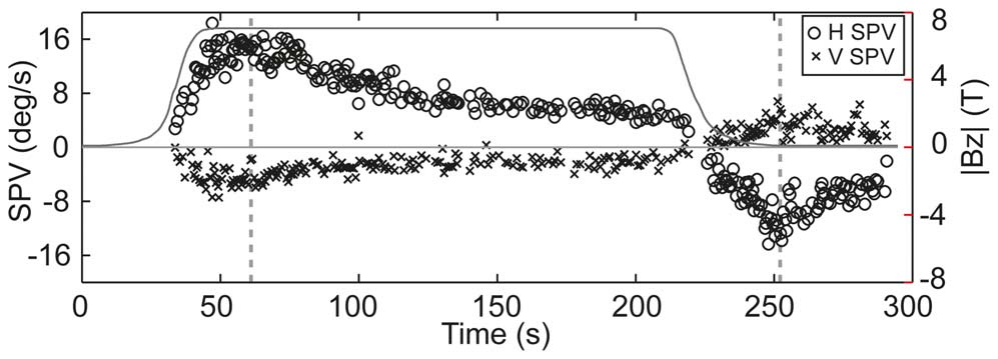
Nystagmus slow phase velocity. Horizontal (circles; Positive = leftward/+Z ocular rotation) and vertical (crosses, Positive =  downward/+Y ocular rotation) slow phase velocity (SPV; left axis) plotted over the magnitude of the Z magnetic field (B_Z_; grey; right axis) during a trial in experiment 2. The vertical lines indicate times at which the bed stops moving following entry into the magnet and stops moving following exit from the magnet.

All participants with eye movement recordings (experiment 1, n = 14; experiment 2, n = 13) developed clear nystagmus with a leftward horizontal slow phase component (+Z ocular rotation) when pushed into the bore of the magnet. The nystagmus direction reversed upon exit from the magnet, a feature which was particularly evident following long-duration exposures (e.g. Fig 3). In some participants, a vertical component was also apparent but this was typically smaller and possibly included a non-magnetic-field contribution. Specifically, nystagmus with very slow (< 2 deg/s) downward drifts (+Y rotation) of the eye were sometimes apparent at very low field strengths, even at baseline (|Bz| ∼ 0.1T) prior to first entries to the high field. The cause of this was not clear. Upon exposure to the high field, the direction of any vertical component varied between participants.

Twenty-four of the 25 participants in this study reported at least one perception of non-veridical motion when exposed to the magnetic field. Descriptions given by participants were coded according to the presence of rotational and translational components. A summary of the incidence of these perceptions is provided in table 2. The most salient and consistent feature of the perceptual reports that was present in almost all participants was rotation in the horizontal plane. This was typically a long-lasting perception of continuous motion. Superimposed on this, some participants experienced periodic, instantaneous resetting of their internal representation of their orientation relative to the long axis (Z) of the room. The phase of this resetting varied between participants, but it ranged between every 45−180 deg of perceived rotation. Sometimes, perception of rotation initially manifested as travelling along a curvilinear path (perhaps rotation coupled with veridical perception of bed motion) prior to perception of pure rotation. With one exception, the direction was always perceived as a –X direction of rotation inside the magnet (cf, Fig 1B). Some participants reported this as their head rotating to the left, some as their legs rotating to the right. We did not routinely ask participants to attend to the perceived centre of rotation, but when sometimes asked post-trial, estimates varied between the centre of the head and the navel. The exception to the simple uni-directional nature of the rotation was a single case in experiment 1 who described a somewhat bizarre perception of motion along a winding path (i.e. with direction changes). In experiment 1, we did not instruct participants to attend to perceptions upon withdrawal from the field, but in experiments 2 and 3, a long duration perception of horizontal plane rotation was typical upon withdrawal and was always in the opposite direction to that experienced inside the magnet.

**Table 2:** Incidence of non-veridical motion perceptions at neutral head positions.

	Experiment 1	Experiment 2	Experiment 3
**Horizontal-plane/roll rotation (about X-axis)**	86%	84%	83%
**Pitch rotation (about Y-axis)**	8%	0%	0%
**Yaw rotation (about Z-axis)**	36%	26%	33%
**Translation**	25%	0%	0%

We parsed motion perception descriptions, given by participants, into different components (cf, Fig 1B). The descriptions sometimes included multiple components (for example, brief yaw rotation followed by longer-lasting horizontal plane rotation). Whenever this occurred, we included all components rather than just the dominant one. However, we excluded components suspected of being veridical. For example, a few participants described a perception of travelling round a bend when bed movement was occurring. We felt this probably involved perception of rotation coupled with veridical perception of translation and so was only coded as containing a rotational and not a translation component. After-effects upon withdrawal from the magnetic field do not contribute to this table.

As shown in table 2, perceptions other than horizontal plane rotation were uncommon. Although their time course was not formally investigated, these perceptions were typically of short duration compared to the perception of horizontal plane rotation and sometimes occurred prior to the onset of the horizontal plane rotation. Sometimes they were only reported for initial entries (scout entries or practice trials), and not subsequent entries. The presence of a reversed direction upon withdrawal from the field was not typical. Perception of rotation about Y or Z axes were sometimes static (i.e. a tilt) rather than a perception of motion, and were of inconsistent direction (about half the instances of perceived yaw rotation were in +Z direction, and about half were -Z).

Non-motion perceptions were rarely reported. In experiment 1, they consisted of pressure on the head (n = 2), phosphenes (n  = 1), metallic taste (n = 1), and perception of a high pitched noise (n = 1).

### Onset field strengths for nystagmus and vertigo

We focused on onset of horizontal nystagmus, specifically the time a left (+Z ocular rotation) slow phase component became apparent. Figure 2a shows detected onset of horizontal nystagmus and non-veridical motion perception with respect to field strength in a representative participant. Perception onset occurred at a substantially higher field strength than nystagmus onset for all participants (Figure 2b; nystagmus onset = 1.67±0.58T; perception onset = 5.14±1.08T). There was a significant correlation between the two onset field strengths (Fig 2c; Pearson r = 0.72, p = 0.008). Two participants were excluded from the nystagmus onset data set: S6 was excluded because of the atypical presence of a rightward (-Z ocular rotation) slow-phase nystagmus at low field (which reversed during entry) and S10 was excluded due to excessive blinking, making onset time unclear.

### Quantitative characteristics of eye movements and perception during long-duration exposure

Pupil tracking for the purpose of ocular slow-phase velocity quantification was possible only for 8 participants (example in fig 3). Our purpose here is only to demonstrate the directional properties and persistent nature of nystagmus inside the magnet. The mean horizontal slow-phase velocity during the first 20 s at 7T was 7.7±3.9 deg/s (positive = leftward/+Z ocular rotation) and mean vertical slow-phase velocity was −0.4±2.3 deg/s (positive = downward/+Y ocular rotation). The slow-phase velocity during the final 20 s at 7T was 4.8±2.3 deg/s (horizontal) and −0.6±2.6 deg/s (vertical). Thus, nystagmus lasted the full duration of exposure to the magnetic field albeit with a decline in magnitude.

**Figure 4:**
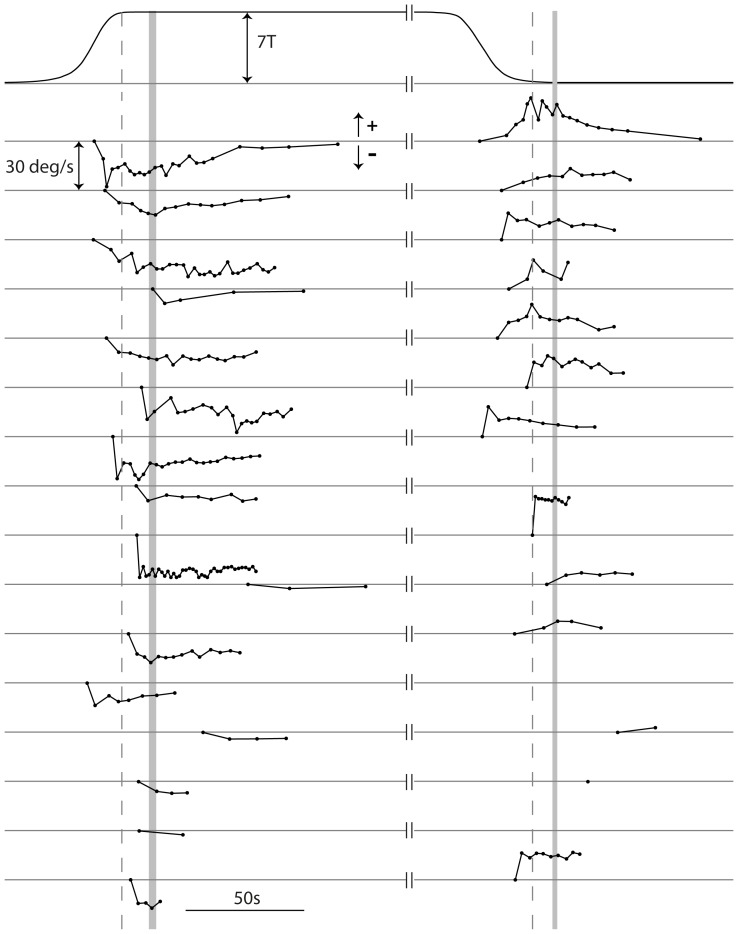
Perceived rotation velocity during long-duration exposures. Solid curve at the top shows group average (relative to alignment events) B_Z_ magnitude experienced by the head. Traces below show perceived rotation velocities for participants who perceived horizontal plane rotation in experiment 2 (16 of 19). Each dot represents time of switch press. Perceived horizontal velocities (upward = positive X, downward = negative X) are plotted relative to individual zero base-lines. The first button press is set to 0 deg/s, although the perceived speed is indeterminate at that point. Individual perceived velocity plots are vertically offset and arranged from top to bottom in descending order of duration of in-phase button presses. Plots have been split horizontally with each part aligned to different events represented by vertical dashed lines (left: aligned to time head reaches 7T during in-phase; right: aligned to time head reaches 0.2T during out-phase). Vertical shaded segments represent 95% confidence interval of the end of bed movement time relative to the alignment events.

Sixteen out of 19 participants experienced perception of horizontal plane rotation in experiment 2. During online logging, participants ignored resetting events (see qualitative descriptions section above) and only used the switches to report the underlying motion. The on-line logging of perceived rotation by these participants is shown in figure 4, with group summary statistics in table 3. The reversal of perception upon withdrawal from the field is clear from these data. During the in-phase, most participants (n = 13) started reporting rotation before the bed stopped moving (i.e. prior to the shaded bar in Fig 4) and some (n = 6) started reporting rotation prior to arriving at 7T (i.e. whilst still experiencing spatial and temporally varying magnetic field; prior to the dotted line). While a few participants had relatively short-lasting perceptions (bottom of figure), most (n = 13) reported rotation for at least 15 s, and up to 90 s, after the bed had stopped moving in the spatially homogenous 7T field (group average = 42 s, table 3).The rotations reported during the out-phase were generally also long-duration responses that persisted well beyond the point at which the magnetic field had dropped to a near zero level (i.e. after dotted line in figure 4; average = 35 s, table 3). Durations (P<.01) and magnitudes of the peak and mean velocities of perception (P<.05) were statistically smaller for the out-phase than the in-phase (table 3).

**Table 3:** Group summary data on perception of horizontal plane rotation during experiment 2.

	In-phase	Out-phase	Out-phase subset
N	**16**	**16**	**12**
B_Z_ at first button press (T)	6.3±1.2	-	1.1±1.3
Final button press relative to end of bed movement (s)	42±23	-	26±16
Final button press relative to start of homogenous (in) or near-zero (out) field (s)	55±22	-	35±16
Duration of button presses (s)	50±25	30±26 **	40±21
Mean velocity (deg/s)	−11±6	8±6 *	11±5
Peak velocity (deg/s)	−15±9	12±9 *	16±7

The table is restricted to the 16 of 19 participants who experienced perception of rotation. Duration of button presses is the time interval between the first and final switch presses. The time of final switch is also reported relative to the end of bed movement in each phase as well as relative to the time the magnetic field becomes homogenous during in-phase (7T) or near-zero (<0.2T) during out-phase. Mean and peak velocities were calculated from button-press data. During the out-phase, three of the 16 participants did not report any perception of rotation and one only pressed the switch once; they were assigned values of zero for out-phase measures in the middle column. The right column shows data just from the subset of participants who pressed the switch more than once during the out-phase. Statistical comparisons between in-phase and out-phase were conducted for duration, and *magnitude* of peak, and mean velocity at the n = 16 sample level (column 1 vs column 2). * P<.05, ** P<.01

The average magnetic field at which the first button press occurred in experiment 2 (∼6T, table 3) tended to be higher than average perception onset signalled verbally in experiment 1 (∼5T). We suspect this is because extra judgement time is required before the participant is confident enough to start signalling rotation direction using the switches, compared to simple verbal indication of the onset of non-specific vertigo. However, a t-test restricted to the 9 participants common to the two experiments did not strongly support a significant difference (experiment 1, 5.1±1.0 T; experiment 2, 6.2±1.5T; p = .08).

### Supplementary trials

Some additional neutral-head-position trials were performed in subsets of participants in experiment 2.

1) Three participants were pushed slowly into the magnet feet first. On these trials, the direction of perceived rotation was opposite to the direction experienced during head first entry (i.e.+X rotation direction during in-phase and –X rotation direction during out phase).

2) Seven participants were pushed rapidly through the field (approximately 5 s from beginning to end of bed motion). During these trials, the rapid movement saturated the bed encoder so we do not have a record of instantaneous bed motion. However, we estimate peak rate of change of field was of the order 10 T/s. None of the participants reported a clear transient vertigo while moving rapidly through the field. In all participants, the prolonged perception of rotation started a few seconds after the bed became stationary at 7T (verbally described at the end of the trial). After withdrawal from the field, a prolonged reversed perception of rotation was experienced commencing a few seconds after returning to the start position. In 3 participants who used button presses to log the perception, the button pressing commenced, on average, 10 s after the bed became stationary. As noted earlier, the commencement of button pressing may represent a slight over-estimate of onset time. Averaged across these 3 participants, duration (s) and magnitude (deg/s) of perception of rotation (in-phase: duration = 69, mean velocity = −14, peak velocity = −19; out-phase: duration = 37, mean velocity = 9, peak velocity = 13) was similar to that experienced during their slow entry trials (in-phase: duration = 68, mean velocity = −13, peak velocity = −19; out-phase: duration = 50, mean velocity = 10, peak velocity = 17).

3) Three participants were moved slowly to 7T and then immediately withdrawn without dwelling at 7T (approx. 35 s at>1.5T during these entries) whilst using switches to log perceived rotation. All participants reported perception of rotation in the –X rotation direction during entry. During exit, all participants continued to report rotation in the same direction for a few seconds while still exposed to the magnetic field. Only one participant experienced reversed perception, which commenced at 0.2T, but it was clearly of much shorter duration (7 s compared to 50 s) than when he underwent long-duration exposure.

### Effect of head yaw

**Figure 5:**
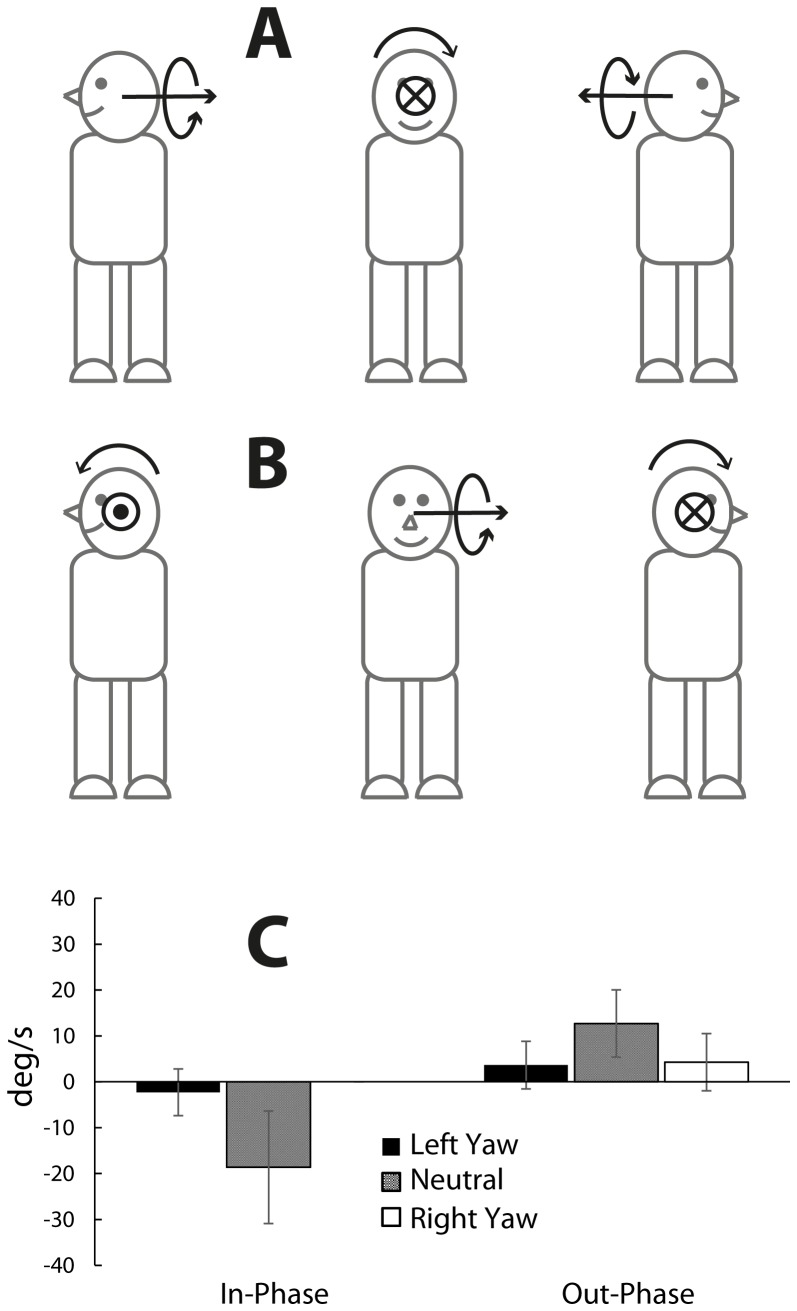
Hypothetical and measured effects of head yaw orientation . **A** and **B** are views from above participant lying supine on bed. Coordinate systems as in Figure 1B **A)** A signal of rotation about the head X-axis (illustrated here as a –X rotation vector) signals rotation in the horizontal plane (about lab X) when the head is neutral, and rotation in the vertical plane (about lab Y) when the head is turned in yaw. Note, the direction of the rotation vector in the vertical plane is reversed for head left vs head right yaw orientation. **B)** A signal of rotation about head Y-axis (illustrated here as a +Y rotation vector) signals rotation in the vertical plane (about lab Y) when the head is neutral, and rotation in the horizontal plane (about lab X) when the head is turned in yaw. Note the direction of rotation in the horizontal plane is reversed for head left vs head right yaw orientation. The head Z-axis will not change in body/lab coordinates as a consequence of head yaw orientation. **C)** Effect of head yaw orientation on mean±SD peak perceived horizontal plane rotation during in-phase and out-phase (Friedman's Test: in-phase, p<.05; out-phase, p<.05). When the reporting switches were being used, no participant (n = 5) reported perception of rotation during right yaw in-phase. However, one participant did report horizontal plane rotation during the equivalent preceding trial without switches, as did a participant who was excluded because of nausea. In both cases the direction was negative.

When the head is in a neutral position, the clear perception of horizontal plane rotation suggests the magnetic field evokes a vestibular signal of rotation about the head negative X-axis. In contrast, the direction of nystagmus suggests a vestibular signal of rotation about the head negative Z-axis (i.e. opposite direction of nystagmus slow phase). A possible reason for this discordance is that perception, unlike nystagmus, is biased to the horizontal plane because apparent rotation in other planes would have greater conflict with reality. When the head is turned 90 degree in yaw, the orientation of the symmetrical magnetic field relative to the head is not affected. Thus, the evoked vestibular signal would stay the same in head coordinates. However, in this position, a rotation signal about the head X-axis now signals rotation in a vertical plane (rotation about lab/body Y) rather than the horizontal plane (Fig 5A). If perception were biased toward the horizontal plane, this perception of vertical plane rotation would be absent or smaller than the perception of horizontal plane rotation in neutral head position.

This was tested in experiment 3. In this experiment, the measured bed movement and field exposure characteristics for the trials conducted with the head turned in yaw (data not shown) were not significantly different (Friedman's test, p>.05) to those reported in the neutral position trials (table 1, experiment 3). Measured head yaw orientation was 84±5 degrees for left yaw and −84±4 for right yaw. With the head turned in yaw some participants experienced perception of rotation in a vertical plane (about the lab Y-axis). The direction of this perception was dependent on direction of the head turn: In left-yaw condition, 4 participants experienced a perception involving the legs lifted above the level of the head; in right-yaw condition, 3 participants experienced a perception of the legs being below the head. These directions were in agreement with those predicted by apparent –X head rotation vector (compare rotation vector in fig 5A for head left vs head right). Unlike horizontal plane rotation in the neutral condition, this perception of vertical plane rotation was generally weak and did not consist of a clear continuous rotation. Instead it was generally described as a small static tilt. This is in agreement with the notion that perception was biased toward signals of horizontal plane rotation. However, there was a single participant who provided an exception. For the head right trial, this participant experienced the perception of large rotational motion in the vertical plane coupled with perception of rotation in the horizontal plane. The experience was nauseating so the trial was aborted early.

Experiment 3 also provides a test of whether the evoked signal contains a component of rotation about head Y-axis. If it does, it would be expected that when the head is turned in yaw, participants would experience perception of horizontal plane rotation and the polarity of the perception would reverse for head left vs head right yaw (compare fig 5B head left and head right, Y rotation vector). The reported perception was strongly attenuated for head yaw trials compared to head neutral trials (fig 5C). Although it was not entirely abolished, any perception of rotation was always of the same polarity for both head yaw positions (thus, not supporting the existence of a significant component of rotation about head Y). This residual horizontal plane rotation during the head yaw trials might have been due to imperfect head positioning i.e. not equal to 90 degree.

## Discusssion

Eye movements recorded in the current study confirm observations of Roberts et al [7]. Mainly horizontal nystagmus was evoked when participants were slowly pushed into the static 7T magnetic field, peaking shortly after arrival at 7T, persisting (with some decline) while participants were stationary in the homogenous centre field, and then reversing direction upon withdrawal from the field. Most participants also experienced a clear perception of horizontal plane body rotation that lasted for about a minute. Upon withdrawal from the field, the perception of rotation re-emerged, this time in the opposite direction.

### Vertigo due to continuous stimulation by the static magnetic field

To explain continuous nystagmus when stationary in a static homogenous magnetic field, Roberts et al [7] proposed that the field interacts with spontaneous, resting ionic current flow in labyrinthine endolymph to induce Lorentz forces strong enough to deflect semicircular canal cupulae. According to this hypothesis, a magnetic field will impose a stimulus to the cupulae that is dependent on magnetic field magnitude and direction [7], [10]. Unlike previously considered mechanisms of magnetic field-evoked vestibular stimulation [4], this mechanism does not depend on movement in magnetic fields, time-varying magnetic fields, or magnetic field gradients. A constant magnetic field (present when stationary) can be considered analogous to a constant angular acceleration stimulus (constant force on the cupula) and a gradually increasing or decreasing magnetic field (as during slow travel into and out of the bore) can be considered analogous to increasing/decreasing angular acceleration.

The dominant vertiginous response in the current study is compatible with this mode of action. First, the rotational nature of the perception strongly implicates involvement of the semi-circular canals. Second, reversal of the direction of perceived rotation for feet-first vs. head-first entry (uniformly present in the subset of participants in which this was tested) is in line with dependence on magnetic field direction. Third, perception of rotation was not only present during bed movement, but was present when stationary in the central homogenous magnetic field of the bore. Although, unlike nystagmus, perception of rotation did not last for the entire duration of magnetic field exposure, this discrepancy in persistence is not unexpected. Studies have shown that during constant angular acceleration, perception of rotation disappears over a time period of the order of one minute despite partly decayed, but continuing nystagmus [8]. This also occurs during constant galvanic vestibular stimulation [c.f., 11,12], another stimulus thought to be analogous to constant angular acceleration [12]. The response decay appears to be due to adaptation to continuous vestibular input. The hallmark of adaptation is the reversal of the response when the stimulus is removed, and so this interpretation is compatible with the reversal of nystagmus and the re-emergence and reversal of perception of rotation upon removal from the magnetic field seen in the current study. Identical behaviour is seen upon termination of constant angular acceleration [8] and constant galvanic vestibular stimulation [11], [12]. It is expected that if stimulus duration is shortened so too is the amount of adaptation and the reversal of the response upon stimulus termination will be attenuated or abolished. Consistent with this, trials performed in a subset of our sample showed that the reversal of perception upon withdrawal from the magnetic field was abolished or attenuated when dwell time in the field was shortened. Why perception apparently decays at a faster rate than nystagmus during continuous semi-circular canal stimulation is beyond the scope of the study, but one possibility is that perceptual and oculomotor adaptation to vestibular input involves different processing within the brain. Another possibility, not mutually exclusive, is that the threshold for perception is higher than nystagmus.

In a number of studies of continuous vestibular stimulation, the group-averaged perceptual response gradually builds up and then decays [8], [12], [13]. In the current study, we report individual responses. The perceptual response profile of some participants showed signs of a gradual build up and decay (e.g. 2^nd^ from top in fig 4) while in many others there was little modulation of the magnitude of the response (aside from some waxing and waning) coupled with an apparent abrupt termination to the perception (e.g. 6^th^ from top). One reason may be that participants found it difficult to accurately grade perceptual magnitude using this mode of reporting. Another reason may be that some participants have high perceptual thresholds relative to the maximum magnetic field strength experienced in this study. However it is worth pointing out that studies that have considered individual perceptual responses to pure continuous angular acceleration stimulation have noted significant waxing and waning of individual responses, particularly when the stimulus involves ramp acceleration [14].

Upon fast entry to the magnet, there was an apparent delay of several seconds between end of bed movement and onset of perception (albeit not precisely established). Delay between onset of stimulation and onset of perception of rotation is a known aspect of continuous angular acceleration, with an inverse relationship between stimulus intensity and perceptual latency [15, p.27]. With the proviso that there is risk in comparison of absolute perceptual magnitudes between studies and between subjects, it is worth noting that there is compatibility between the evoked magnitude of perception of rotation (average peak speed of around 10 deg/s in the current study) and delay in onset of perception. Thus, angular accelerations of approximately 0.5-1 deg·s^−2^, which have been reported to evoke peak perceptions of rotation of around 10 deg/s during long duration stimulation [8], are associated with around 3-7s delay in detection of rotation [15].

There was discordance between the plane of perception and the plane of nystagmus that must be accounted for if they are to be explained by a unitary mechanism. In the neutral head position, the mainly leftward horizontal nystagmus slow phase (+Z ocular rotation) implies a vestibular signal of rotation about the head Z axis (specifically, -Z rotation vector) while the perception implies a vestibular signal of rotation about the head X axis (specifically, -X rotation vector). The lack of an oculomotor response about the head X-axis may have been more apparent than real. Rotation in this direction corresponds to torsional eye movements, which may have been present but could not be detected using our video-based method of eye-movement recording. The lack of a clear perceptual response about the head Z-axis can be explained by interaction between canal input and other sensory inputs. In the neutral head position, any component of rotation about the body Y or Z axes would mean a change in body orientation with respect to gravity. This would be in conflict with other sensory input signalling the body's orientation with respect to gravity, e.g. otoliths and cutaneous inputs. In contrast, there will be relatively little conflict with a signal of rotation about a vertical axis, and therefore little resistance to the formation of perception of rotation in the horizontal plane. Such a conflict with gravity receptors was apparent when the head was turned in yaw, which should have transformed the horizontal plane illusory motion into a continuous vertical plane rotation but failed to do so.

The nystagmus and perceptual evidence both suggest an absence of a significant component of rotation about the head Y-axis. First, there was no consistent vertical nystagmus in the neutral head position. Secondly, when the head was turned in yaw such that any signal of rotation about the head Y-axis would now signal rotation in the horizontal plane (therefore amenable to a perceptual response), significant rotation with directionally appropriate properties was not observed (experiment 3).

Thus, based on the combined nystagmus and perceptual observations we suggest there is a single illusory rotation vector evoked by the static magnetic field, the orthogonal components of which drive the dominant oculomotor and perceptual responses. Specifically, a magnetic field parallel to the Z-axis of the head induces a signal of rotation that has a net vector in the mid-sagittal plane of the head with the direction of the vector components being in the –X and –Z directions. The precise orientation of this vector is difficult to calculate based on the current observations since we do not know the relative gains of the eye movements and the perceptual rotation speeds.

### Alternative mechanisms

In the previous section we argued that the perceptual observations are compatible with a mode of action that is dependent purely on continuous stimulation by the static magnetic field, in common with observed horizontal nystagmus. However, since the perception of rotation and the horizontal nystagmus represent orthogonal components of vestibular input, it is also possible that they may be driven by entirely independent processes. Glover et al [4] described three possible mechanisms of magnetic field-evoked vertigo. These mechanisms required head movement within the magnetic field (magneto-hydrodynamics), time-varying magnetic fields (induced electric currents), or direct effects of magnetic field gradients (diamagnetic susceptibility). Such conditions are experienced when the bed is travelling into the bore, but not when stationary in the central homogenous field. Thus, these mechanisms would produce transient, rather than continuous vestibular input. A number of earlier studies tended to favour transient mechanisms dependent on time- or space- varying magnetic fields as primary causes of vertigo when patients enter MRI scanners [1], [2], [4], [6]. Although some survey-based studies did report the presence of vertigo after bed motion had stopped, the incidence and magnitude was considerably lower than during movement [1], [2], [6]. However, if interpreted with vestibular adaptation and multisensory weighting in mind, these observations are not incompatible with the notion of continuous stimulation by the static magnetic field. In these studies, visual input and the use of standard beds may have meant that veridical motion cues, and perhaps some adaptation to a significant baseline field exposure, severely curtailed development of illusory self-motion perceptions. This may have given the misleading impression that vestibular stimulation is transient, occurring only when moving during entry and exit. In the current study, we performed experiments in the absence of vision and used a custom made bed which was constructed to give considerably smoother travel compared to conventional scanner beds. The custom bed also allowed us to start entry from a baseline field strength of approximately 0.1T rather than 1T, which meant that any adaptation to the magnetic field at baseline was reduced, and perception of vestibular input was given a good chance to develop and persist.

Nonetheless, it is known that relatively short duration periods of angular acceleration (i.e. <10 s) of sufficient magnitude can produce rather long (> 30 s) after-sensations of rotation [13], [16] that may be compatible with the average 50 s duration of perception in the current study. Furthermore, although the diamagnetic susceptibility mechanism is incompatible with the reversal of perception upon exit [4], the magnetohydrodynamics and induced current mechanisms are both compatible with this as well as with reversal of perception during feet-first entry. We cannot totally exclude these possibilities, but a number of observations and inferences lead us to favour the more parsimonious explanation that perception and nystagmus share a common mechanism involving non-transient vestibular stimulation: 1) There was no statistical difference between the in-phase and out-phase duration of exposure to dB/dt, whilst average bed velocity and peak dB/dt tended to be slightly higher during the out-phase. This would suggest that the magnitude of any transient stimulation should be approximately the same, or even slightly larger, for the out-phase compared to the in-phase. In contrast, the magnitude and duration of the perceptual response was statistically lower for the out-phase; 2) Long duration after-rotations to transient vestibular stimulation seem to be associated with much stronger peak perceptual experiences than those reported in the current study. For example, Okada et al [17] report decay of perception of rotation lasting approximately 40 s following a velocity step. The peak perceived rotation velocity was approximately 60 deg/s, in contrast to approximately 10 deg/s in the current study. 3). When transient stimuli are of sufficient intensity to produce long-lasting after-rotations, the literature does not suggest substantial delays in onset of perception. For example, Sinha et al [18] use a velocity step of sufficient magnitude to evoke perception for approximately 40 s. There was less than 1 s average delay before perception was noticed, as reported elsewhere for angular accelerations greater than 4 deg·s^−2^ [15]. In contrast, during fast entry into the magnet there was a delay of several seconds (relative to end of bed movement) before perception of rotation was noticed. 4) Theoretical calculations of magnetohydrodynamics related to head movement and induced current would suggest that head movement and dB/dt properties experienced in the current study are insufficient for vestibular stimulation [4].

### Onset of nystagmus and vertigo

The onset of vertigo (∼5.1T) was at a magnetic field 3 times higher than the onset of horizontal slow phase eye movements (∼ 1.7T). It is important to recognise that these do not represent thresholds of vertigo and nystagmus. Since we measured onset during uninterrupted movement into the bore, any delays in onset of these responses relative to vestibular stimulation will mean the onset fields represent overestimates of response thresholds. This is of practical relevance because most current MRI machines are 3T or less, and it may be noted that other studies have reported that vertigo can sometimes be experienced at 1.5T [1], [6]. The rapid entry trials described in this paper suggest there is indeed significant delay in the onset of the perceptual response and it is reasonable to suppose that such a delay will be larger than any delay in the nystagmus response, thereby influencing the difference between onset fields for vertigo and nystagmus. A comprehensive test for response thresholds would involve moving participants to particular field strengths and waiting several seconds to see if responses develop. Nevertheless, it is notable that the relative difference in our onset fields for nystagmus and vertigo is not very different to difference in thresholds when vestibular stimulation is induced by actual rotation. For example, Seemungal et al [19] reported threshold of perception at an angular acceleration 2.3 times that at which nystagmus was detected. Furthermore, the significant correlation between the onset fields for nystagmus and vertigo is compatible with the view that they arise from a common mechanism.

Another pertinent point is that overt thresholds do not necessarily equate to magnetic vestibular stimulation thresholds. Thresholds of nystagmus and vertigo are influenced by both vestibular input and its central processing, as well as by methodological limitations. If the proposed Lorentz force mechanism of stimulation is correct, it may be that at the vestibular transducer/afferent level, there is no meaningful threshold to static magnetic field-evoked stimulation, analogous to stimulation by angular accelerations [20].

## Conclusion

Vertigo evoked during exposure to the static magnetic field of an MRI scanner was compatible with a mode of action involving continuous, non-transient stimulation of the semi-circular canals, in common with magnetic-field evoked nystagmus. Observations were also in line with the recently proposed Lorentz force hypothesis. Whilst the current study does not entirely exclude alternative mechanisms dependent on movement through the field, these mechanisms seem less likely. Continuous vestibular input due to static magnetic fields have potential implications for the interpretation of central activation patterns during functional MRI. Thus an understanding of the 3d nature of this vestibular input could be important. If it is true that nystagmus and vertigo are both driven by continuous input from the static magnetic field, their collective spatial properties suggest that with the magnetic field approximately parallel with the superior-inferior axis of the head (as is typical during head MRI), the evoked vestibular signal contains both head roll and head yaw components.
